# Transactions of the Buffalo Obstetrical Society

**Published:** 1885-02

**Authors:** 


### TRANSACTIONS OF THE BUFFALO OBSTETRICAL SOCIETY.

*Stated Meeting, Dec. 23, 1884.*

The President, Dr. W. W. Potter, in the Chair.

Dr. Thomas Lothrop read a paper on " Rest during the Menstrual and Puerperal Periods."

He began by referring to the alternate conditions of activity and repose, as being essential in the economy of nature, and important factors in the problem of life; and showed their importance to the physician in analyzing causes and effects as pertaining to morbid phenomena.

He next proceeded to consider the local concomitants of the menstrual epoch, such as congestion or hyperæmia of the ovaries, uterus and mammæ; the exaltation of nervous force, both local and general, and the maturation and escape of the ovum. He contrasted the physiology of the past and the present, and proceeded to discuss the changes in the female economy which take place at puberty, signalized by the establishment of the menstrual flow.

The perodicity of this newly-established function was referred to as its mysterious and inexplicable peculiarity; it is accompanied by an increase of all the vital forces of the body, and new activities are set in motion. Then comes prolonged rest, when the organs are quiescent, until the phenomena are repeated by the ripening of another ovum. The proportion of women who suffer morbid disturbances during menstruation was shown, according to reliable authority, to be forty-six per cent. How can this be explained of a function which is strictly physiological? The conditions, he asserted, which were imposed upon woman through her present social environment, depreciated vital force; and the standard of health was often affected by antecedent causes, which were created by the faulty hygienic conditions of infancy, childhood, and adolescence. Can we not, he continued, further explain the morbid phenomena which statistics unfold as accompanying the performance of the menstrual function, such as pain due to obstruction, congestion or uterine hyperæsthesia, and the manifold hystero-neuroses, on the theory that muscular development and nutrition are lost sight of in the fondness for artificial excitement, which unduly stimulates the nervous activities of the sex, and which build up a physique morbidly sensitive to every external disturbance, and finally develop weaknesses which cause sterility and premature decay? The conditions of the uterus and its appendages during menstruation are, he asserted, often upon the limits of pathology, if not precisely morbid.

Pain, he said, may depend upon—

1. Imperfect power of resistance in the nerve centers, rendering them unduly susceptible to the increased vascular tension.

2. Disproportion in the development of the uterus, resulting in organic defects in the genital canal.

3. Imperfect nutrition and development from prolonged celibacy or sterility; or, the sudden suppression of the menstrual flow; or, the accidents of pregnacy or parturition.

Again, the mucous membrane, at the menstrual period, is swollen to the extent of 0.118 to 0.236 inch, and of almost pulpy consistency; showing that the uterus is in that peculiar condition in which any disturbance of the vascular equilibrium may easily convert a natural physiological engorgement into a pathological state. The morbid processes once set up are quite likely to persist, and gravitation further aids in the development of the various displacements to which the organ is liable.

The essayist then proceeded to show how rest tends to enable the vital forces to regain lost vigor, to allay morbid irritability, and to restore the equilibrium between the nervous and muscular forces where the nervous is excited at the expense of antagonizing force. The neuroses of menstruation, such as hysteria, convulsions, paroxysms of insanity, headache, cramps, catalepsis, etc., he classified as cases of reflex irritation upon the nerve centers, whose powers of resistance have been abnormally diminished. For the want of control of nervous and muscular action in these highly sensitive organizations, rest is the *sine qua non* at each menstruation. In strictly normal menstruation nature takes care of herself; but, in the very large per cent. of cases which are not natural, and which have passed beyond the confines of physiology into the domain of disease, rest fulfils an indication which can be practically applied with incalculable benefit. He further elaborated the meaning of the term rest, as here used, as being absolute physical and mental repose.

He next took up the subject in its relations to the puerperal state, where the morbid phenomena are varied, and often sadly fatal. Here, he asserted, the responsibility of the physician is very great, even the weightiest of all his professional duties.

High civilization and refinement render women less able to bear the shock of parturition; pain and difficulty in parturition are probably artificial consequences of these social conditions. In South Africa, Iceland and among savage tribes there is less suffering and greater fertility than in the higher states of civilization. Accepting these data, and recognizing that the same antecedent causes which lead to disturbances in the menstruating woman, may affect the puerperal state, it is interesting to inquire whether the complications and difficulties which surround the latter may not be arrested or diminished by hygienic measures? In the changes in the constituents of the blood, such as the diminution of the red corpuscles and albumen, and the increase of white corpuscles and fibrin; also in the decrease of its density, and the increase of fatty phosphorized matter, may be found the *raison d'etre* of inflammations, of septic infections and the allied diseases. The changes in the respiratory organs were alluded to, particularly with reference to the increased consumption of carbon during pregnacy, and the query propounded: Is the arrest of tubercular disease in the gravid woman, and its rapid progress after parturition, due to the strict solidarity between the uterus and the respiratory function, the first supplementing the other, as declared by Barnes? The dynamic state of the circulation during pregnacy was referred to: First, superior arterial hyperæmia; and, second, inferior venous hyperæmia; and the relations these may bear to hypertrophy of the heart, and to venous obstructions in the lower extremities, pointed out. The changes in the glandular system were next considered; enlargement of the thyroid and the spleen, together with the changed dimensions and relations of the liver, the salivary, sebaceous and sudoriparous glands were referred to, and the conclusions drawn from this brief review of the changes in the organs and fluids of the body, was that the border-land of pathology is near at hand in the puerperal state; that nature is jealous of any imprudence in her victims, and that the *vis conservatrix naturæ* fails to guard the sex from the dangers of this important period.

The local changes in the pelvic organs were next considered. The measurements of the uterus at the full period after expulsion of the fœtus were stated to be from 12 to 19 centimeters; at the expiration of two weeks it is reduced to 9 to 12 c., the length of the cavity of the non-puerperal woman being 6 to 7 c. The weight of the uterus immediately after parturition is from 22 to 24 oz., and even 30 oz. At the end of the first week it is reduced to 19 to 21 oz.; second week, 10 to 11 oz., and at the end of the third to 5 to 7 oz., the normal weight, 1 ½ to 2 oz., being gradually reached at the end of two months, when not impeded by disease. The failure of this process of reduction to normal size and weight—subinvolution—is one of the great hindrances to puerperal convalescence.

The change in size and weight of the uterus is brought about by, first, action and tonic contraction of the muscular fibres; second, by absorption and excretion. If the first is interfered with, we have resulting blood-stasis, disposition to hemorrhage, and often feeble glandular action and impaired nutrition. The regeneration of the new mucous lining is frequently delayed. Subinvolution of the vagina is often a sequence of parturition of no small moment.

For the prevention of the various pathological sequences of pregnancy and parturition, as well as for the promotion of early convalescence after delivery, the essayist urged that puerperal women be encouraged to take physical and physiological rest, more or less prolonged, according to the conditions surrounding each case. The authorities cited by the essayist in support or elucidation of the various asseverations, histological, pathological and physiological, were numerous and the quotations trite. He concluded with the following summary:

1. That rest during and subsequent to menstruation, under the artificial conditions of the environment of the sex in this age, is indicated: first, as a preventive to disease, and, second, as a prudential measure for physical suffering.

2. That rest or repose, after parturition, is indicated for the restoration of the organism to its *status quo*.

3. That the traditional custom of strictly observing the lying-in month has the sanction of experience.

4. That the conditions of the sex under the influence of barbarism, or the imperfect civilization of Iceland, cannot be accepted as a standard to guide the profession in our more advanced civilization.

5. That the altered conditions of the organism under the influence of menstruation and pregnancy present abundant data to sustain the physiological and prudential necessity for rest for self-reparation.

*Discussion*—Dr. J. W. Keene, in opening the discussion, remarked that it was quite possible to over-estimate the importance of rest. He regarded the performance of the menstrual function as a physiological one, and the organs involved in its office are but obeying the natural law of habitual rest after a period of activity, which characterizes the functions of all the organs of the body. The heart beats and rests; the brain acts and rests; the activity of the stomach is periodical; the phe nomena of menstruation obey the same law. There was danger, he thought, in the enjoining of physical repose in these cases as a routine practice, in laying the foundation or habit of chronic invalidism. We find, in the wealthier classes, a great tendency to indolence and a condition of chronic invalidism. They like to be sick. We should, therefore, be careful how we enjoin upon a girl, at a period when she is full of morbid fancies attendant upon the establishment of new functions, a period of a week's rest.

We all agree, he continued, with the main features of the paper, that in some cases rest is imperatively necessary. In other cases it seems to me not only unnecessary, but is liable to do harm. Ordinary care and the avoidance of unusual exertion should, no doubt, be carefully enjoined, and the young female should be taught the significance of this function. Right here is a great field for missionary work before the physician. Inculcate in mothers the necessity of teaching their daughters the significance of these things, so that a girl will know how to care for herself during the menstrual week, and avoid undue and unnecessary exposure. It is interesting, said he, to notice the grouping together of the phenomena of menstruation, and those before and after parturition. Baudelocque, so far as he knew, was the first to voice this idea, and who held that every menstruation was a miniature abortion, in which an ovum was lost or did not go to full term, and only lacked the more or less important factor of impregnation.

He wished to speak of one or two points further: First, the elucidation of the modification of phthisis by the pregnant condition. He supposed that to be one of the ideas of the older writers which was now exploded; that the latest teaching is that the existence of pregnacy serves to hasten the development of phthisis, even in cases where it had not yet appeared. Exceptions to this rule could be explained on other grounds. Second, physicians very generally agree, now-a-days, as to the propriety of rest during the puerperal state at certain periods, notably in those where abortions have been found to be especially imminent. Just how long rest should be enforced after parturition was still a question. The ninth or tenth day does well as routine practice; some can get up, however, on the fifth, while others cannot do so without risk until the fifteenth day. The guide should be the general condition of the female, and especially the condition of the uterus. If it has undergone involution, so that it has escaped above the pubes, there seems no good reason why a woman should be kept in bed two or three weeks subsequent to that time.

Dr. W. D. Greene said that he was impressed with the remarks of the essayist as to women in the olden time having no pain during parturition. This he could hardly accept if the lower animals furnished an analogue to judge from. He also thought that there were certain conditions during menstruation when a woman would be benefited by the erect posture and moderate exercise; for instance, when there was a narrowing of the canal which served to dam up the fluids. Here the law of gravitation might prove beneficial.

Dr. F. H. Potter said that the vital point in this matter was, not what a barbarian woman can do, but what a civilized woman *does* under her present environment. A few years ago he had occasion to look into the subject in one of its branches, and, in doing so, he was led to tabulate the work required of the pupils at Welles and Elmira colleges. He found the time devoted to mental work was out of all proportion to the time given to rest. They arose early in the morning, and studied and walked, or worked in the gymnasium until nine o'clock at night. Something was going on all the time, without pause, or rest, or cessation from duties. It seemed to him that just at this critical time in life, when nature required all the strength she had to properly develop the new function, it was abominable for persons pretending to be educators to insist that young women should do this great amount of work without proper periods of rest.

Dr. W. S. Tremaine remarked that he had been favored with opportunities of observing parturition, and its effects in the civilized and the uncivilized. Menstruation and parturition in females who live in a natural state are perfectly normal processes. They are not perfectly normal processes in civilization. One reason for this difference, he said, was to be found in the fact that civilization makes an immense demand upon nerve tissue. When the tissues of the body are perfectly balanced, that constitutes health; when an extra demand is made upon any one tissue, a tendency to disease is produced. In regard to uterine displacements, as alluded to by the author, he remarked that the evil of the present day seemed to him to be too great a tendency to localism. There could be no question of the value of rest, and he referred to Dr. Wier Mitchell as having taught us valuable lessons on this subject. Among Indian women menstruation amounts to nothing more than expectoration, as far as its effects on the economy were concerned; and parturition was purely a physiological process. If there is some reserve force in the nerve tissue, the evils attendant upon the performance of these functions will be avoided. Hence, he believed in the propriety of rest, for it served to store up strength in the exhausted nerve tissues.

Dr. H. D. Ingraham was in favor of rest during the menstrual period, especially in the case of young girls; did not believe a woman would properly develop unless she took considerable rest during the period in which she was changing from girlhood to womanhood. He did not believe the brain and the ovaries could be developed at the same time without material harm to one, and the ovaries generally suffered most. He thought our boarding schools and female colleges turned out a larger proportion of invalids than of healthy girls. With regard to Dr. Goodell's practice in getting his puerperal women up early, while it is true he does so, yet he guards them with a careful hand and keeps them very quiet after they do get up.

Dr. George E. Fell said that this question of the schools was an important one, and physicians should take hold of it in an interested and earnest way, with a view to prevent the physical disasters from over-work on the part of young girls. There is no question, he said, but that there are radical defects in the methods of teaching, not only in the higher schools and colleges for females, but in our common schools, and these evils are having an influence for harm upon the health of the future mothers of the land. As to the question of rest during the puerperal period, he believed that in many instances it was very necessary; while, on the other hand, there were many conditions of the puerperal woman where activity seems to benefit her. He thought that the intelligent physician would govern these various conditions according to their necessity, and that no other rule could be laid down.

Dr. R. L. Banta found that the subject had been so well and thoroughly gone over, that he had very little to add; but it was his privilege to see all sorts and conditions of puerperal women, some of whom would get the family dinner on the second day after accouchment, and would make a good recovery, while others he had seen in bed three or four months, yet they, also, finally got well. Now he was of the opinion that Dr. Fell had struck the key-note of the whole matter when he asserted that this was a question for the individual judgment of the physician in charge, who should either permit or interdict activity according to the necessities of the particular case in hand. He even thought it was rest for some women to get up soon after labor.

Dr. Lothrop said, in closing the discussion, that there had been too little opposition to his views for him to make extended remarks in reply; but one thing he would amplify a little was in relation to the effect of pregnancy upon phthisis. He had witnessed the phenomena mentioned in the paper too often to feel doubtful as to the fact that the progress of the malady was often arrested by gestation, and as often had he seen these cases proceed to a rapidly fatal termination after parturition.

